# Evaluating system usability of mobile augmented reality application for teaching Karnaugh-Maps

**DOI:** 10.1186/s40561-022-00189-8

**Published:** 2022-01-15

**Authors:** Rubina Dutta, Archana Mantri, Gurjinder Singh

**Affiliations:** Chitkara University Institute of Engineering and Technology, Chitkara University, Punjab, India

**Keywords:** Augmented reality, Learning environment, System usability, Immersion, Engineering education

## Abstract

The education system evolves and transforms towards interactive and immersive learning tools in this digital age. Augmented reality has also evolved as a ubiquitous, robust, and effective technology for providing innovative educational tools. In engineering education, many abstract concepts require technological intervention for conceptual understanding and better instructional content. While learning through the immersive tools, system usability has great importance in terms of effectiveness, efficiency, and satisfaction. Effectiveness refers to users' accuracy and completeness in achieving defined goals; efficiency relates to expended resources about the precision and completeness with which users achieve their objectives; satisfaction deals with a positive attitude towards using the product. If the system fails to provide good usability, it may cause adverse effects such as increasing stress, lacking necessary features, increasing the users' cognitive load, and negatively impacting the student's motivation. In this study, two mobile augmented reality (MAR) applications were developed as an instructional tool to teach the students about Karnaugh maps in the digital electronics course. The first application is a Keypad-based MAR application that uses a keypad matrix for user interaction and the second application is a Marker-based MAR application that uses multiple markers to solve K-Map for producing an optimum solution of the given problem. An experimental study was conducted to determine the student's opinion of the developed MAR applications. The study was designed to determine the system usability of the two MAR applications using the System Usability Score (SUS) and Handheld Augmented Reality Usability Score (HARUS) models. 90 engineering students participated in the study, and they were randomly divided into two different groups: keypad-based group and Marker-based group. The keypad-based group included 47 students who had hands-on experience with a keypad-based MAR application, whereas the marker-based group included 43 students who had hands-on experience with multiple marker-based MAR applications. The experimental outcomes indicated that the keypad-based MAR application has better SUS and HARUS scores than the marker-based MAR application which suggests that the keypad-based MAR application has provided better user interaction.

## Introduction

Learning is an ongoing process for everyone. Traditional teaching approaches depend on the information learned from books and teachers and then applied to solve real-world problems (Dutta et al., 2020). The education system continues to evolve and transform towards a collaborative learning model. Students learn through social networks, collaboration, and immersion in digital spaces to seek, share and create information for self-realization (US Department of Education & Office of Educational Technology, 2017). Universities emphasize incorporating Information and Communication Technology (ICT) tools in the classroom to fulfill learners' demands. Learning through such kinds of environments and tools is known as e-learning (Thamarana, 2016). E-Learning is a form of technology in which learning materials are distributed digitally through the Internet, thereby promoting learning by removing time, distance, and socio-economic barriers (Gohiya, n.d.). HTML pages with embedded images and videos make up most e-learning systems. They are all two-dimensional and lack interactivity (Kumar et al., 2020). Many educators believed that interactivity creates an enjoyable learning environment where students can build interest and use the problem-solving approach towards any real-time problem situation. Ubiquitous learning (u-learning) and mobile learning (m-learning) were used to make the learning more realistic and interactive (Chang et al., 2018). They both use similar tools but may use in different ways. U-learning offers dynamic content and requires a specialized environment, whereas m-learning is flexible to use at any place and offers the scalability feature (Vallejo-Correa et al., 2021). Nowadays, Virtual Reality (VR) and Augmented Reality (AR) are the technologies that provide blended features of u-learning and m-learning. In AR and VR, 3-D dynamic content could be created; students can experience and visualize the 3-D content using smart gadgets such as PDAs, mobile phones, desktops, etc. (Boonbrahm et al., 2016).

The pedagogies mentioned above are innovative in various educational domains such as primary, secondary, K-12, medical, industrial automation, and engineering (Hwang et al., 2016; Prit Kaur et al., 2018). All have their own merits and demerits, but AR has been proved as one of the promising technologies; it provides interactivity, immersion, and instant feedback features. It has also been proved to be a guiding tool for learners to perform different experiments and learn critically about complex real-time problems (Chang & Hwang, 2018). The principal merit of AR is that learners can perform any laboratory-related experiment at any time without spending any money on the costliest hardware equipment.

AR is a technology that overlays virtual objects with real-world objects. It constitutes three main characteristics: the fusion of the physical world and the virtual world, real-time interaction, and 3D registration (Azuma, 1997). Over the last few years, there has been increasing popularity in research interest of AR, as mobile devices such as smartphones and tablets have provided users with much simpler and cheaper access to AR than before. Positive effects of AR technology on student learning, critical thinking, learning motivation, learning experience and collaborative learning, etc. have also been reported in the previous studies (Akçayır & Akçayır, 2017; De Amicis et al., 2018). By considering all such benefits, an AR-based system has been developed for engineering students to learn and solve the complex problems related to the basic electronics and digital electronics course. Students in electronics engineering frequently design circuits and logic. Digital electronics is a key subject for electronics, electrical, and computer science engineers because it helps learners develop their logic-building abilities. Students were able to construct the logic and solve the K-map on their own while solving design challenges using the Karnaugh map (K-map), but they had difficulty identifying the optimal solution out of redundant pairs. Students require the assistance of teachers to validate the logic design because they lack the necessary skills. The following are the common mistakes that student do while applying K-maps:Wrong selection of redundant pairs, Creating wrong logic expressons based on redundant pairs, Unable to create correct AOI logic diagram. Hence, there is a need for a flexible (inside/outside the class) learning environment where students follow the K-map steps to get the correct logical solution. In this study, an AR-based learning environment was developed to address students' issues with K-map learning. Using an AR-based mobile application, students can learn the K-map method step-by-step and get instant feedback from the system at each stage of designing. Also, AOI logical diagrams for any Boolean expression can be developed by interacting with the application.

At the initial stage, mobile augmented reality (MAR) using multiple markers (marker-based approach) was designed to solve 2-variable based Karnaugh-Map (K-Map) problems. Later, mobile-augmented reality using a keypad matrix (keypad-based approach) was designed to solve the same. The marker-based approach consists of multiple markers (as mentioned in Fig. 4) to solve the K-map up to 2-variables. When it comes to 3 or 4 variables, it provides a lot of flickering effect, as a single mobile camera is responsible for detecting multiple markers simultaneously. Hence, a flickering effect produces on the mobile screen and the student is not able to design the logic diagram correctly. To solve this issue, keypad based approach was used that replaces multiple markers with a single piece of a marker. This single marker overlay the virtual information of the keypad matrix on the mobile screen. By selecting the push button, the student can populate the cells of K-Map and could perform necessary steps by clicking the tabs mentioned on the right side of the application (as mentioned in Fig. 11).

### Importance of usability

**Fig.1:**
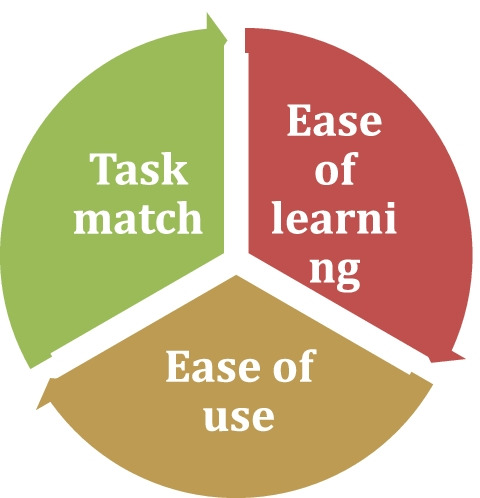
Identifiers of usability

As per the ISO (international standard organization), usability is described as the 'amount to which specific users can use a product to achieve specific goals with quality, effectiveness, and satisfaction in a specific context of use' (Lewis, 2018). The primary identifiers of usability are given in Fig. 1. The ease of learning refers to the effort needed to comprehend and operate a new method (Elfaki et al., 2013). Further, it depends on the user's experience and how easily that knowledge can be mapped into the unfamiliar framework.

The term ease of learning refers to the effort needed to operate a device until the user has fully comprehended and mastered it. The task match relates to how well the information and functions provided by a system meet the user's needs (Elfaki et al., 2013). Along with this, ISO identified three attributes. They are effectiveness, efficiency, and satisfaction. Effectiveness refers to users' accuracy and completeness in achieving defined goals; efficiency relates to expended resources about the precision and completeness with which users achieve their objectives; satisfaction deals with a positive attitude towards using the product (Is et al., 2016). Testing usability of the system is of paramount importance as it tells the missing or complex features of the designed system. Some of the positive impacts of evaluating system usability are:

- *Attention span* In a recent survey, students' attention spans were found to be between 10 and 15 min. In the educational field, AR technology has gained traction because of its immersive learning environment, which includes both virtual and real objects, and the ability to learn by doing (Di Serio et al., 2013; Squire & Jan, 2007; Yilmaz, 2017).
- *Motivation* Students become motivated and show interest in learning complex topics only if they are satisfied with the system they are using (Chang & Hwang, 2018; Lai et al., 2019; Yilmaz, 2017).
- *Process-Validation* A suitable hierarchy should be followed before deploying a new framework for students. Teachers should test the system because their input is critical to the development of a better version. (Kumar et al., 2020).
- *Misconception* Students have the misconception that they are not ready to adopt a new approach to learning because it requires a great deal of effort. If the system is simple to understand and meets the needs of the students, they may be able to improve their knowledge. The more they learn, the better their overall performance will become (Akçayır & Akçayır, 2017; Chang & Hwang, 2018; Singh et al., 2019).

If the system does not meet the needs of its users, it may cause stress, a lack of features, an increase in cognitive load, and a negative impact on the motivation of the student.

### Approaches for measuring system usability

Specific models such as Technology Acceptance Model, Eason Model, Shackel Model, System Usability Scale (SUS), Handheld Augmented Reality Usability Scale (HARUS), Neilson model, and User Experience Questionnaire (UEQ) were identified to evaluate the usability and validity of the system. Table 1 provides the various models, which were used to measure the usability of the system.

**Table 1:** Models used to measure system usability

Models	Sub attributes	References
User Interaction Satisfaction (QUIS)	Screen visibility, terminology, system information, learning factors, and system capabilities	Chin et al. (1988)
Software Usability Measurement Inventory (SUMI)	Global, efficiency, effect, helpfulness, control, and learnability	Cavallin et al. (2007)
Post-Study System Usability Questionnaire (PSSUQ)	Satisfaction, usefulness, information, and interface quality	Lewis (2018)
System Usability Scale	Effectiveness, efficiency, and overall ease of use	Brooke (2020)
People at the centre of mobile application development	Effectiveness, efficiency, learnability and memorability, satisfaction, errors, and cognitive load	Az-Zahra et al. (2019)
Eason Model	Task, user, system	Eason (1991)
Shackel Model	Effectiveness, learnability, flexibility, attitude	Koohang and Ondracek (2005)
Nielsen Model	Learnability, efficiency, memorability, errors, satisfaction	McDougall et al. (2001)
ISO 9241-11	Effectiveness, efficiency, satisfaction	Horton and Leventhal (2008)
ISO 9126	Understandability, learnability, operability, attractiveness, usability compliance	Horton and Leventhal (2008)
Handheld augmented reality usability scale (HARUS),	Comprehensibility, manipulability	Santos and Sandor (2008)

Depending upon the following parameters, such as performance, speed, degree of effectiveness, efficiency, and satisfaction for the user to complete the work, SUS and HARUS models were identified to evaluate the performance of the designed system.

## Research objectives

To calculate the usability of the system following research questions are addressed.

- *RQ1* To determine the system usability of marker-based and keypad-based AR application using the SUS model.
- *RQ2* To compare and analyze system usability of marker-based and keypad-based AR application using the HARUS model.

The structure of the paper is divided into the following sections: "Literature review" section consists of a background of a teaching–learning approach based on AR in the engineering domain; “Design” section incorporates the design of the proposed system and its associated challenges; “Research methodology” section represents the experimental design of the research work; “Result and discussion” section discusses the results using SUS and HARUS scale; “Conclusion” section represents the conclusion of the research work.

## Literature review

In education, augmented reality (AR), virtual reality (VR), and mixed reality (MR) are widely used to enhance students' learning experiences, critical thinking ability, and skills. Numerous research papers have been presented in the literature to examine the effect of AR on learning abilities, motivation, knowledge gain, critical thinking skills, and cognition. The literature revealed that using AR-based interaction methods to teach the idea of logic gates in digital electronics has been attempted, but it involved only abstract concepts of logic gates (Avilés-Cruz & Villegas-Cortez, 2019). A mobile application was proposed to provide immersive and interactive features to the users to enhance learning (Selek, 2020). A desktop-based application was devised to help gain laboratory skills without buying the costliest equipment, such as cathode ray oscilloscope and function generator (Singh et al., 2019). Moreover, for the conceptual understanding of complex topics, a system was designed which helped the user to learn and visualize the system response in different conditions such as damped oscillations, critical response, etc. (Prit Kaur et al., 2018). An AR learning environment was created where students could understand the Arduino Uno programming and their connection with peripherals and also enhanced their hardware skills. As we know, the initial step towards learning a hands-on approach is the understanding of breadboarding. An AR mobile application was proposed where students could learn basic prototyping (Garcia-Sanjuan et al., 2018). Besides, the proposed application acted as a self-guiding tool and provided knowledge to students in every aspect. Table 2 summarises the numerous AR applications, especially in electronics and electrical engineering education.

**Table 2:** AR-based learning environments in electronics and electrical engineering

Engineering discipline	Topic	Approach	Findings	Evaluation Technique	References
Electrical Engineering	Electromagnetic prerequisites	AR based learning environment	Students were able to imagine abstract physics principles and gain a deeper understanding using AR experience	Knowledge test and critical thinking questionnaire	Faridi et al. (2020)
Electronics Engineering	AR to electronics Practice	AR based mobile application	These findings show that incorporating augmented reality into electronic practice has a significant impact on student achievement	Academic achievement of students was analysed	Selek (2020)
Electronics Engineering	Embedded system	AR based table-top learning environment	Before deploying the system on students, system usability was evaluated	SUS is used to measure system usability	Kumar et al. (2020)
Electronics Engineering	Electronic circuit simulation using AR	AR based mobile application	Increase learning efficiency by simulating and moving intangible concepts like current, voltage, and so on into the physical world	Learning quality of teaching was analysed	özüağ et al. (2019)
Electronics and Electrical Engineering	Control System	AR based mobile and tabletop learning environment	For theoretical topics, students rated the ARLE as an efficient learning method. Also, usability of two design variants of the ARLE in terms of manipulability and comprehensibility was discussed	HARUS is used to evaluate system usability	Prit Kaur et al. (2019)
Electronics Engineering	Laboratory equipment (CRO)	AR based tabletop learning environment	The findings of this study indicate that augmented reality (AR) is a suitable technology for creating interactive AR experiences for engineering education	Cognitive load and learning motivation	Singh et al. (2019)
Electronics Engineering	Logic gates using AR	Handheld AR device	The marker less paradigm identifies the ICs and places three objects: IC identification, pins information, and logic diagram information. The AR system's overall evaluation was given a technical efficiency of 97.5	Survey questionnaire was prepared to evaluate system usability	Avilés-Cruz and Villegas-Cortez (2019)
Electronics Engineering	Resistive electrical circuit on breadboard	AR based mobile application	The results of the research show that the system aids the user in comprehending and comparing the theoretical and experimental values of circuit parameters via an AR layer displayed on the smartphone	User experience, perception, and perceived ease of use was evaluated	Garcia-Sanjuan et al. (2018)
Electronics Engineering	Analog and digital communication	AR based mobile application	Students gave feedback that hand-held device cause hindrances in robust equipment handling so it could be improved by using HMD’s	Accuracy and efficiency of the system was calculated	Baloch et al. (2018)

From the literature studies, it has been devised that an AR-based learning system helps the students to learn complex topics through game-based learning (Lin et al., 2011). Along with this, some negative aspects of the technology were also discussed such as students may become cognitively overburdened due to the large amount of information they encounter, the multiple technological devices they must use, and the complex tasks they must complete. In AR environments, when students were engaged in a multi-user AR simulation, they often felt overwhelmed and confused because they had to deal with unfamiliar technologies as well as complex tasks (Wu et al., 2013). So, before deploying the system on a large number of students, its usability must be accessed by the experts or a small group of students. The expert's feedback could help the designer to improve the overall performance of the system such as ease of use, speed, learnability, comprehensibility etc. In the field of mobile applications, there are three types of usability evaluation methodologies: laboratory experiments, field studies, and hands-on measurement. The current work utilized a hands-on measurement methodology in which students used the system and expressed their opinions both qualitatively and quantitatively. They specifically identify the system's strengths and weaknesses, which aids the designer in enhancing the system's performance in the future.

## Design

The Mobile Augmented Reality (MAR) application is used to help the students to learn about K-Map. The MAR application will help the students in determining the optimum solution of digital design problems using K-maps. The MAR application is an active learning platform and a self-guided learning tool that allows the students to solve various digital design problems. Using input data, the framework guides and advises the learner through each step of the K-Map solving process. The following hardware components are used for developing MAR application: Arduino Uno Development Board, HC-05 Bluetooth module, the Keypad-matrix, the paper markers, 9 V battery, breadboard, Logic gates, LEDs, and connecting wires. The following software tools are used for development of MAR application: Unity 3D game engine, Vuforia SDK, and Adobe photoshop. Unity and Vuforia SDKs were used to write the system's scripts. The MonoDevelop IDE's C# script can be used to develop and modify any Unity project. When it comes to creating augmented reality (AR) applications, Vuforia is a software development kit (SDK) for smartphones (such as cell phones). Computer vision is used by Vuforia to quickly and accurately identify three-dimensional (3-D) objects. An Arduino interface is used to verify the physical hardware circuit. Unity 3D Bluetooth Plugin receives the data from the Arduino Uno via the HC-05 Bluetooth module. After building a breadboard circuit with an Arduino interface, the Bluetooth module will send information about its correctness to the Unity 3D Bluetooth plugin. “Correct Connection” or “Incorrect Connection” will appear on the mobile screen display.

### Concept formulation

It covers the basic understanding of K-Map, step-by-step process, rules to solve K-Map, and learning objectives.

#### Background of K-map

**Fig.2:**
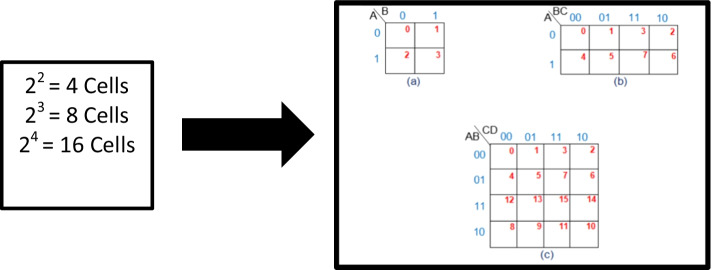
**a** 2 variables K-Map, **b** 3 variables K-Map and **c** 4 Variables K-Map

Digital electronics is a field that studies how to miniaturize circuits by finding the optimum solution. K-Map is a technique that minimizes Boolean expressions and provides a simplified logic design. While applying K-maps, students often made mistakes/errors which cause problems in determining the solution. So, AR is utilized to develop the learning environment for helping the students. K-Map technique is a two-dimensional graphical approach with 'n' variables and 2^n^ cells. Only the position of the single-bit differs between adjacent cells. Figure 2 shows the pictorial view of 2, 3, and 4 variable K-Map.

#### Learning objectives

Following were the learning objectives for students to be able to do after learning through the designed system;

- To implement various logical functions using logic gates by understanding the difference between analog and digital circuits.
- To obtain a basic level of Digital Electronics knowledge and set the stage to perform the analysis and design of complex digital electronic circuits using Karnaugh-Map (K-Map).

### System description

**Fig.3:**
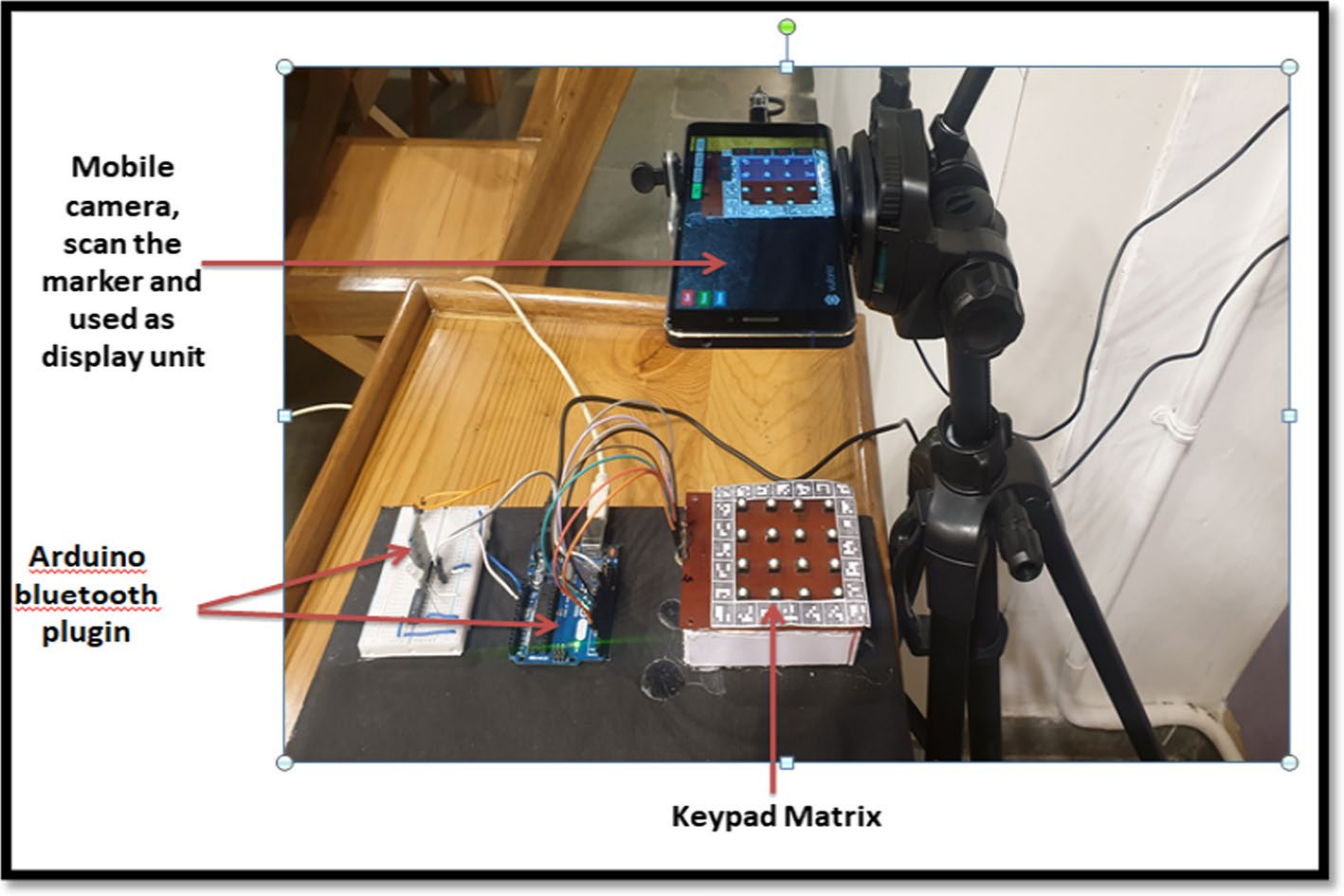
System design for MAR using keypad matrix (hybrid tracking)

**Fig.4:**
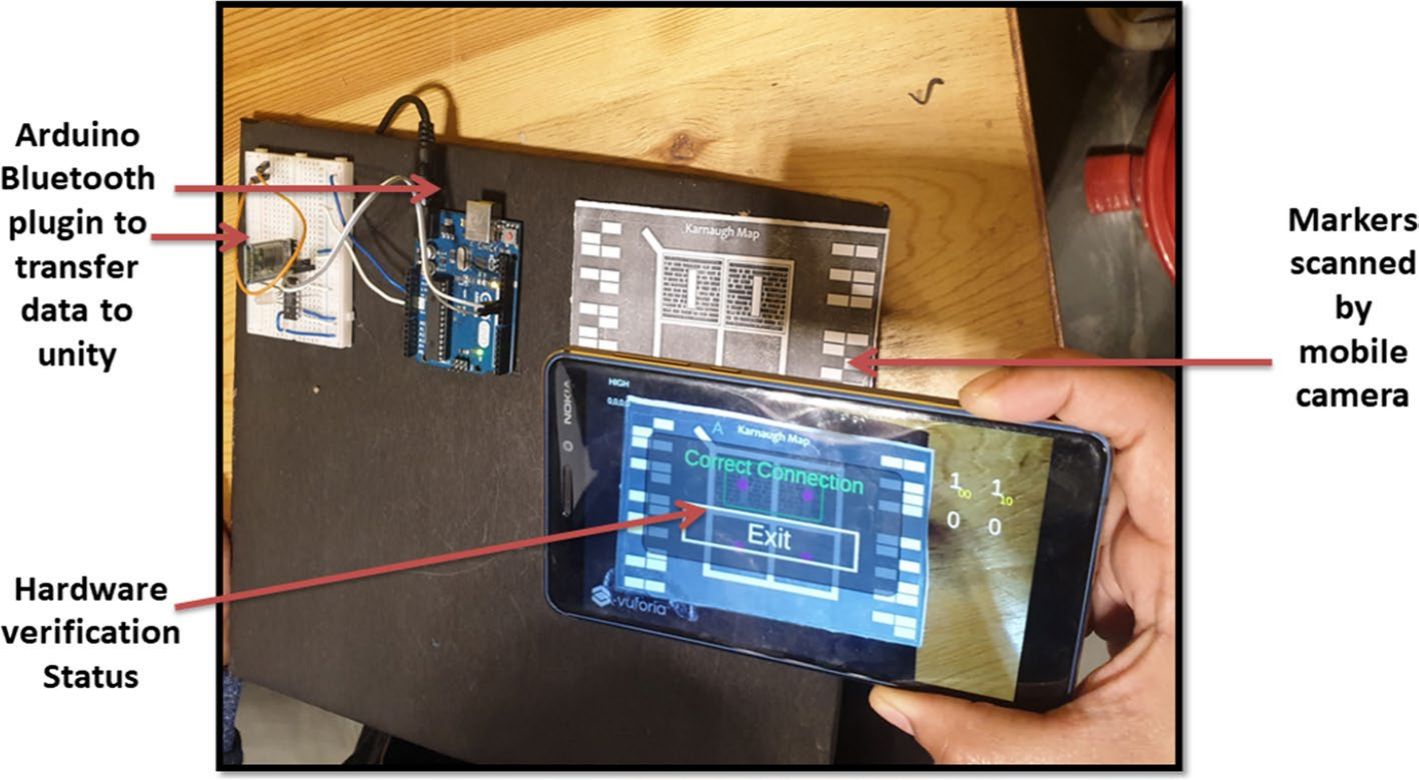
System design for MAR using multiple markers

MAR application was proposed to improve the teaching–learning processes and enable students to assimilate and manage fundamental concepts that make digital systems possible. The students will be able to understand the K-Map and its related process using this system. Figures 3 and 4 present MAR design for 2-Variable K-Map using two different methods, such as keypad-based (hybrid tracking) and marker-based.

One handheld device (mobile/tablet), a keypad matrix, a paper marker, an Arduino Uno, a Bluetooth device, a breadboard, a logic IC, an LED, and connecting wires comprise the keypad-based system. A mobile camera is used to detect the location of the marker, which aids in the display of the virtual keypad on a mobile screen. The student can select the state of K-Map cells (either 1 or 0) using the keypad matrix; the selected state from the keypad matrix will be displayed on the mobile screen via AR technology. After populating the cells, the student can form a pair by clicking on the “Pair tab”; after forming a pair, the estimated equation can be verified by clicking on the “Equation tab”; an AOI diagram can be designed by clicking on the "Diagram tab"; and the output of the AOI diagram can be verified with real-time output (circuit built on the breadboard as shown in Fig. 3) by clicking on the "Verify tab." While transferring the data from the keypad to the mobile screen and verification of virtual output with real-time output, the Arduino Bluetooth plugin plays a significant role. It helps to communicate the information from hardware to Unity 3D software.

The markers-based system employs multiple markers. One large marker defines the cells of K-Map, while small markers populate the cells of K-Map (small markers indicate state 1 in K-Map cell). Figure 4 shows two upper cells displaying the state "1" and two lower cells displaying the state "0." Following the placement of the markers, a mobile/handheld device is used to detect the markers; once the markers have been detected, the user can form the pair by selecting the "Pair formation tab." Once the correct equation is selected by the user then logic diagram (AOI) options will be displayed on the screen. The system performs “connection verification” (virtual output with the real-time circuit) after the user selects the correct diagram. The Arduino Bluetooth plugin is used to verify the connection. It uses Unity 3D to transfer the real-time circuit's final output. If the virtual output status matches the output of the real-time circuit, the message "correct connection" will be displayed; otherwise, the message "incorrect connection" will be displayed.

#### MAR using multiple markers

MAR using multiple markers was designed to solve 2-variable K-Map. The system contained one big marker that represented the structure of a 2-variable K-map (along with a division of 4 cells). Besides, the 2-variable K-map contained 2^2^ = 4 cells with a maximum of four combinations. It also consisted of 4 markers to represent numbers of 1 s and help populate the K-map. The specifications of the system are tabulated in Table 3.

**Table 3:** Specifications of 2-variable K-Map with multiple markers

Software:	Unity
AR SDK:	Vuforia
Platform:	Mobile
Graphics design:	Adobe photoshop
Bluetooth plugin:	Android studio
Hardware components:	Markers, Bluetooth module, Arduino, Logic gates, connecting wires, LED’s
Tracking technique:	Multiple marker based

#### Stepwise working MAR using multiple markers

**Fig. 5:**
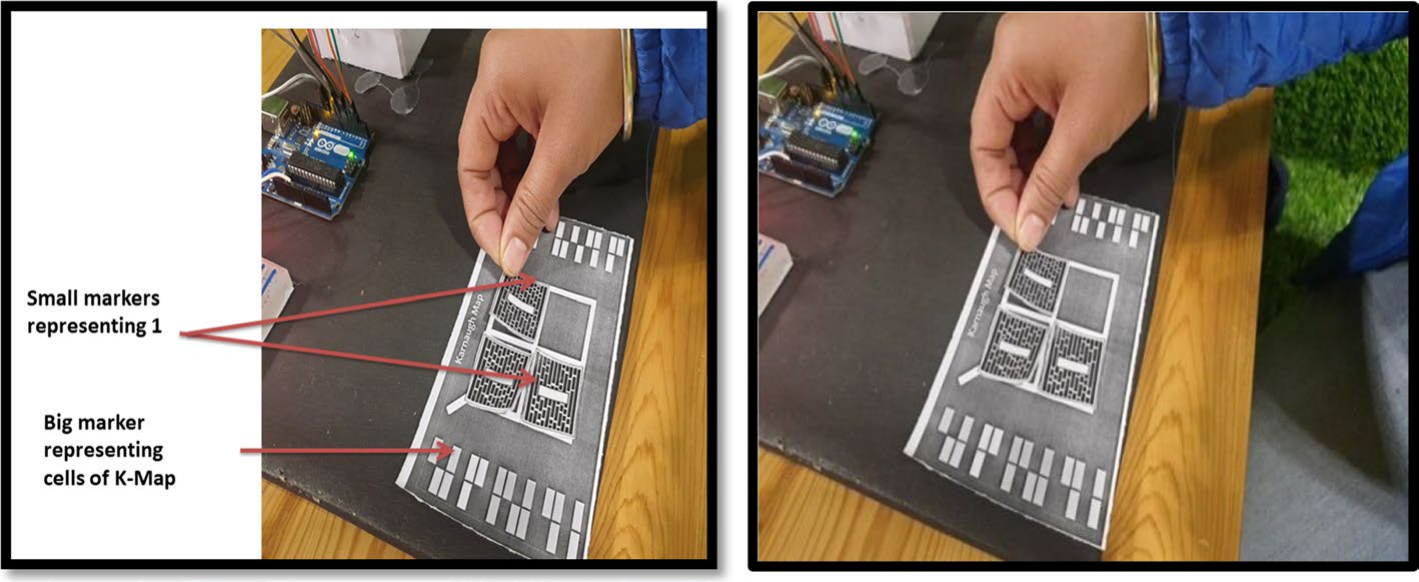
Placement of markers that represented several 1 s

**Fig. 6:**
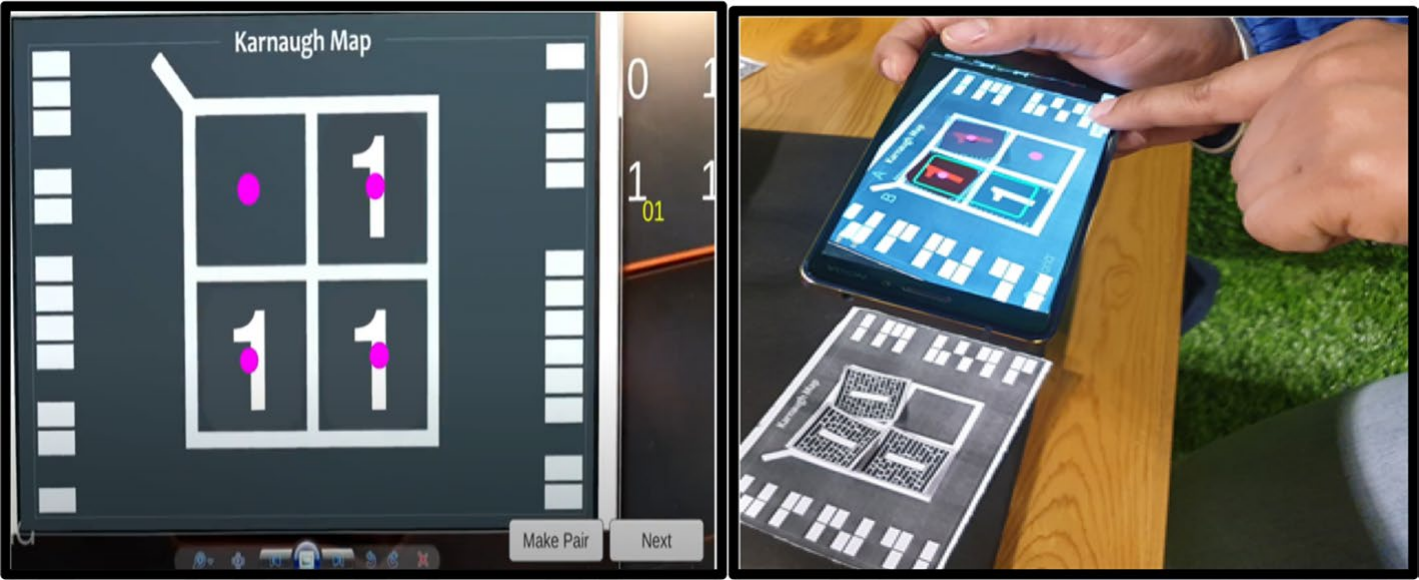
Formation of pairs

**Fig. 7:**
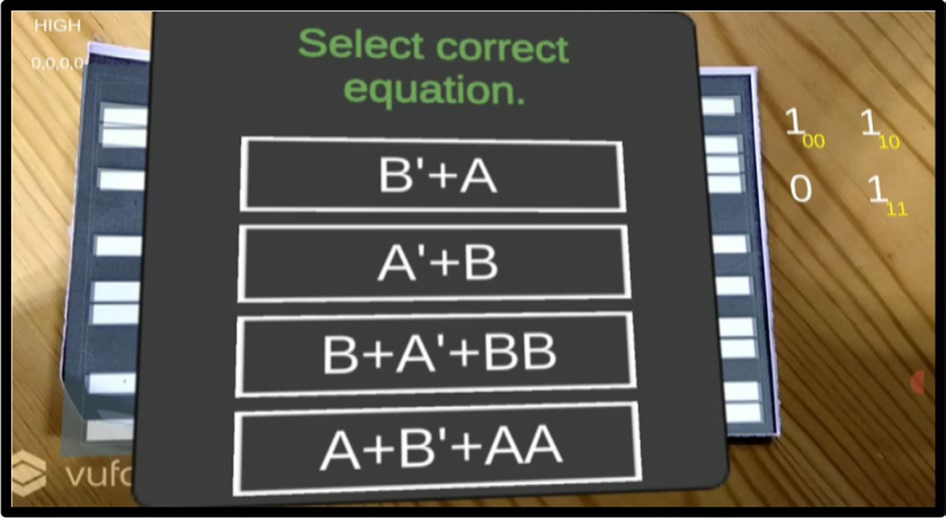
Learner to choose the correct equation

**Fig. 8:**
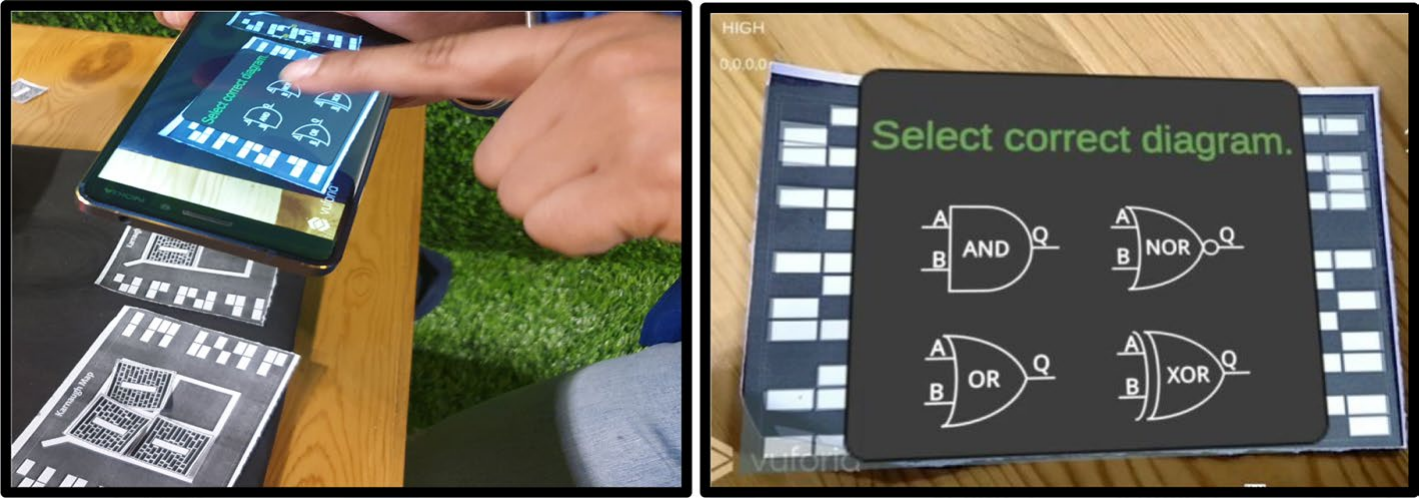
Learner to select the correct logic diagram

- *Step 1* As per the given truth table/problem statement, students were to place the markers at the correct positions. The pictorial view of placing markers is as given in Fig. 5.
- *Step 2* Scan the markers from the mobile application and then form the pair. Click on the "Make pair" tab for pair-formation, as shown in Fig. 6
- *Step 3* After forming the pairs correctly, an equation dialogue box will appear on the screen. The user has to select the correct equation out of four equations, as shown in Fig. 7.
- *Step 4* Once the correct equation is selected, the AOI diagram will appear on screen as given in Fig. 8.

**Fig.9:**
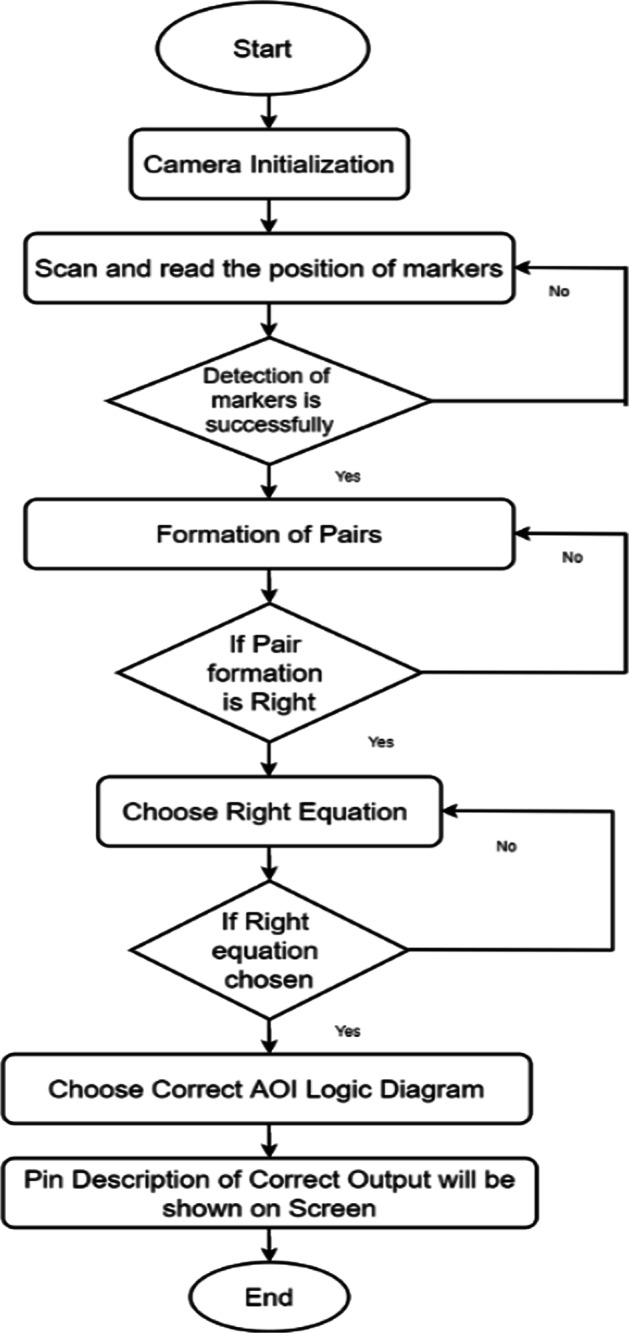
Workflow of MAR using multiple markers

The complete flow of the system is presented with the help of a flow chart diagram, as shown in Fig. 9

#### MAR using keypad matrix

The MAR using a keypad matrix uses a hybrid tracking technique. It consists of a 4 × 4 matrix of push buttons along with one marker. This single marker helps to overlay the virtual information of push buttons in a real-time environment. Using this keypad matrix, users can perform 2, 3, and 4 variable K-Map. Through this, users can interact with the system and can solve any problem based on K-Map. The specifications of the 2-variable MAR using a keypad matrix system are given in Table 4.

**Fig. 10:**
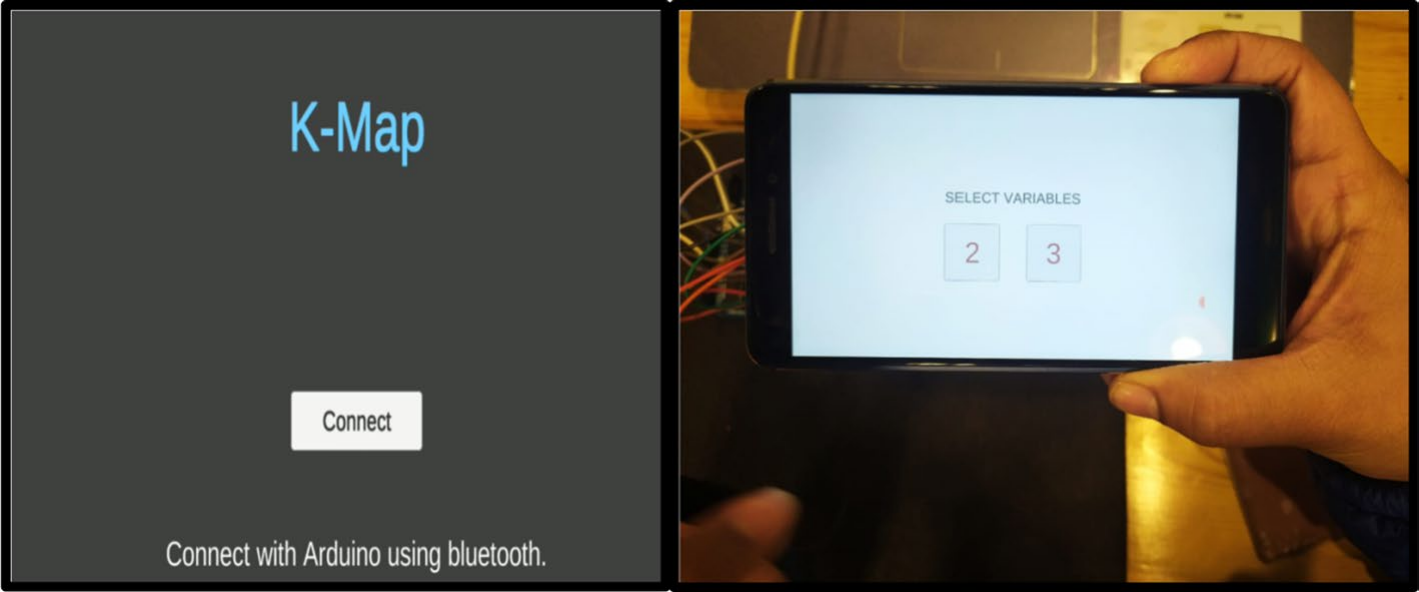
Connect the device and select variables

**Fig.11:**
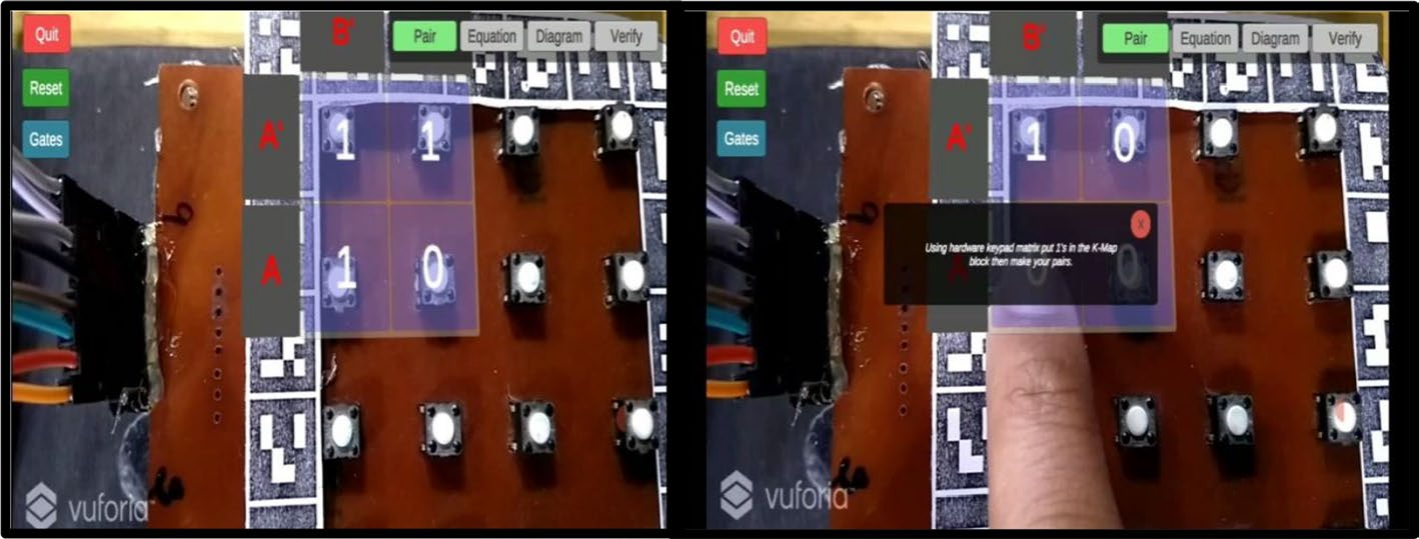
Populate K-Map after changing state of pushbuttons

**Fig.12:**
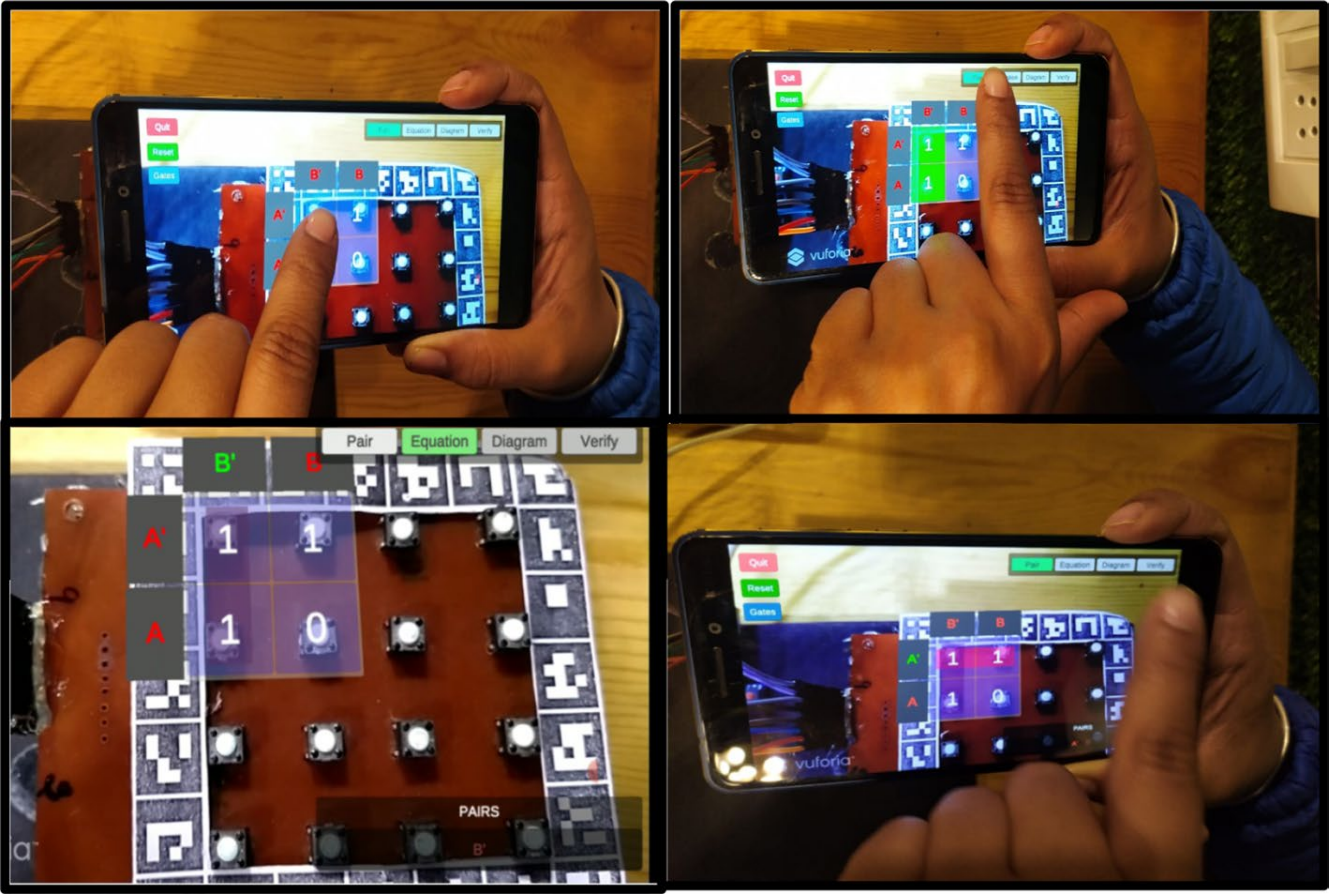
Pair formation of selected 1’s

**Fig.13:**
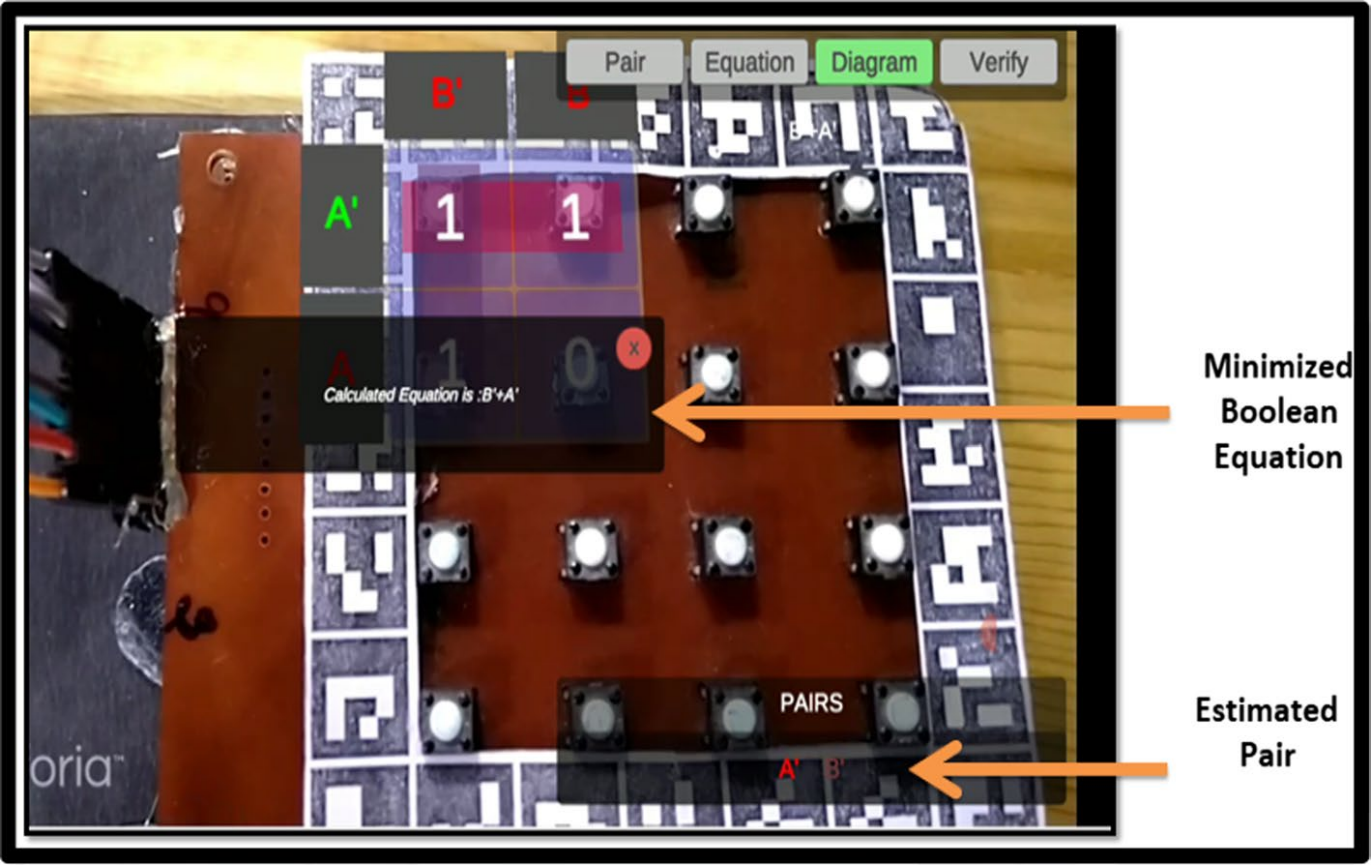
Represent the correct and minimized equation

**Fig.14:**
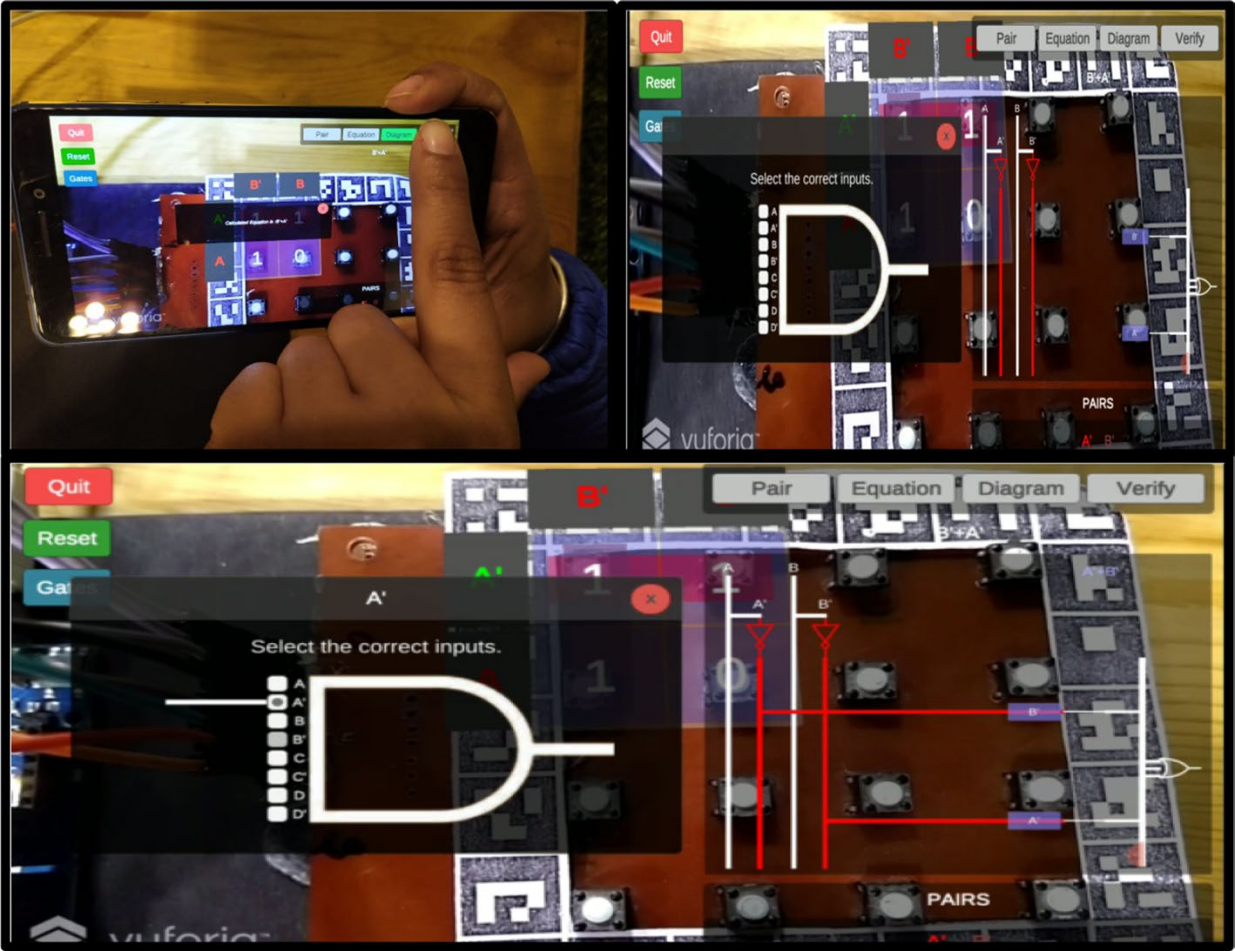
AOI logic diagram

- *Step 1* An android plugin was designed to transfer the data of push buttons on Unity. Initially, the application identifies active Bluetooth devices and tries to connect with them. Afterward, the user can select the variables on which s/he wants to work. Figure 10 depicts the initialization of the MAR.
- *Step 2* After selecting the 2-variable tab, the 4 selected cells will be displayed on the screen (refer to Fig. 11). By manual selection pushbuttons, users can populate the K-Map.
- *Step 3* Once K-Map is populated, then form the pair by clicking on 1's. Once a pair is formed, it shows that pair's common literals with the highlighted green color (refer to Fig. 12). This helps to teach the students how to calculate common literals from the formed pairs.
- *Step 4* While forming pairs, common literals were presented at the bottom of the screen. The user can click on the "equation" tab mentioned on the screen to verify the equation. Once clicked, it shows the system's optimal solution, and the user can understand where they are performing wrong (refer to Fig. 13).
- *Step 5* Lastly, the user will form AOI diagrams. Once s/he clicks on the “Diagram’ tab, the AOI diagram appears on the screen. By selecting the virtual pushbuttons on the AND gate, the user can complete the logic diagram (refer to Fig. 14).

**Table 4:** Specifications of 2-variable K-Map using keypad matrix

Software:	Unity
AR SDK:	Vuforia
Platform:	Mobile
Graphics design:	Adobe photoshop
Bluetooth plugin:	Android studio
Hardware components:	Keypad matrix, Bluetooth module, Arduino, IC’s, connecting wires, LED’s, Breadboard
Tracking technique:	Hybrid tracking (vision and single marker)

**Fig.15:**
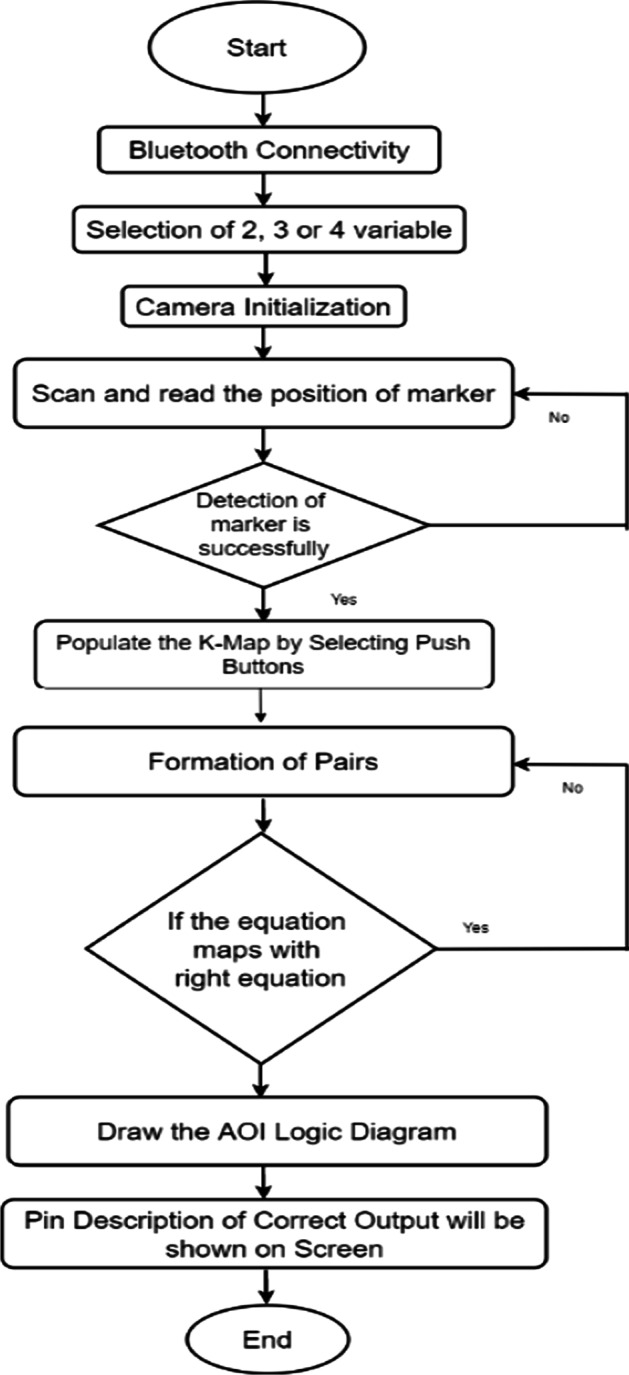
Workflow of MAR using keypad matrix

The complete flow of the system is also presented with the help of a flow chart diagram (refer to Fig. 15).

### Design challenges

One of the major challenges faced while developing MAR with multiple markers was: simultaneous detection of multiple markers. For 2-variables, tracking 4 markers was manageable, but for 3, 4 variables, it was not easy to track multiple markers. This problem was overcome by replacing the whole system with keypad matrix buttons. Another most significant challenge faced was while transferring keypad matrix data to the Unity3d MAR application. An android plugin was developed to enable data transfer between Unity3d and Arduino. Challenges were also faced while designing the mobile application, locating augmented content exactly with keypad matrix buttons, pair visualization, and creating dynamic diagrams. Table 5 presents the design challenges faced while designing MAR using two different approaches.

**Table 5:** Design challenges faced while developing MAR applications

Parameters	Mobile application using multiple markers	Mobile application using keypad matrix
Detection of markers	Difficult to read multiple markers simultaneously using mobile camera	Easy to detect as it consists of a single marker and uses hybrid tracking technique
Transfer of bits	No need	Difficult to transfer
Flickering effect	More	Less
Overlaying of virtual data on real object	Easy	Difficult
Pair visualization	Only highlight the selected pair	Shows the common literal along with selected pair
AOI diagram representation	Shows static diagram	Shows a dynamic diagram

## Research methodology

The research methodology adopted for the study consists of participant details, data collection instruments, and experimental procedures.

### Participants

In this study, 90 engineering students aged from 18 to 20 years voluntarily participated. Before conducting the activity, participants were explained the study's objectives so that they must actively participate in the experiment. All the participants were arbitrarily divided into two groups: the keypad-based group and the Marker-based group as mentioned in Table 6.

**Table 6:** Participant details

Groups	Male	Female	Total
Keypad-based group	22	25	47
Marker-based group	23	20	43
Total	45	45	90

### Data collection and instrument

This study used two scale models: System Usability Scale (SUS) and Handheld Augmented Reality Usability Scale (HARUS), to evaluate system usability. The present work aims to measure the system usability of two different MAR applications developed as a teaching aid. The survey included 10 questions related to ease of use of the method and confidence on the Likert scale of 1 to 5. Here, "1" corresponded to "Strongly Disagree," and "5" corresponded to "Strongly Agree". (Brooke, 2020). The Cronbach's alpha of the survey questionnaire was 0.88 showing internal consistency of the questionnaire.

HARUS emphasizes the perceptual and ergonomic issues related to the application. HARUS incorporates two major components: manipulability—the ease of handling the AR system, and comprehensibility—ease to read the information presented on screen. The HARUS questionnaire incorporates 16 questions that focus on the general problems found in any handheld device (Santos & Sandor, 2008). The Cronbach's alpha of the survey questionnaire was 0.78 showing internal consistency of the questionnaire.

### Experimental procedure

**Fig. 16:**
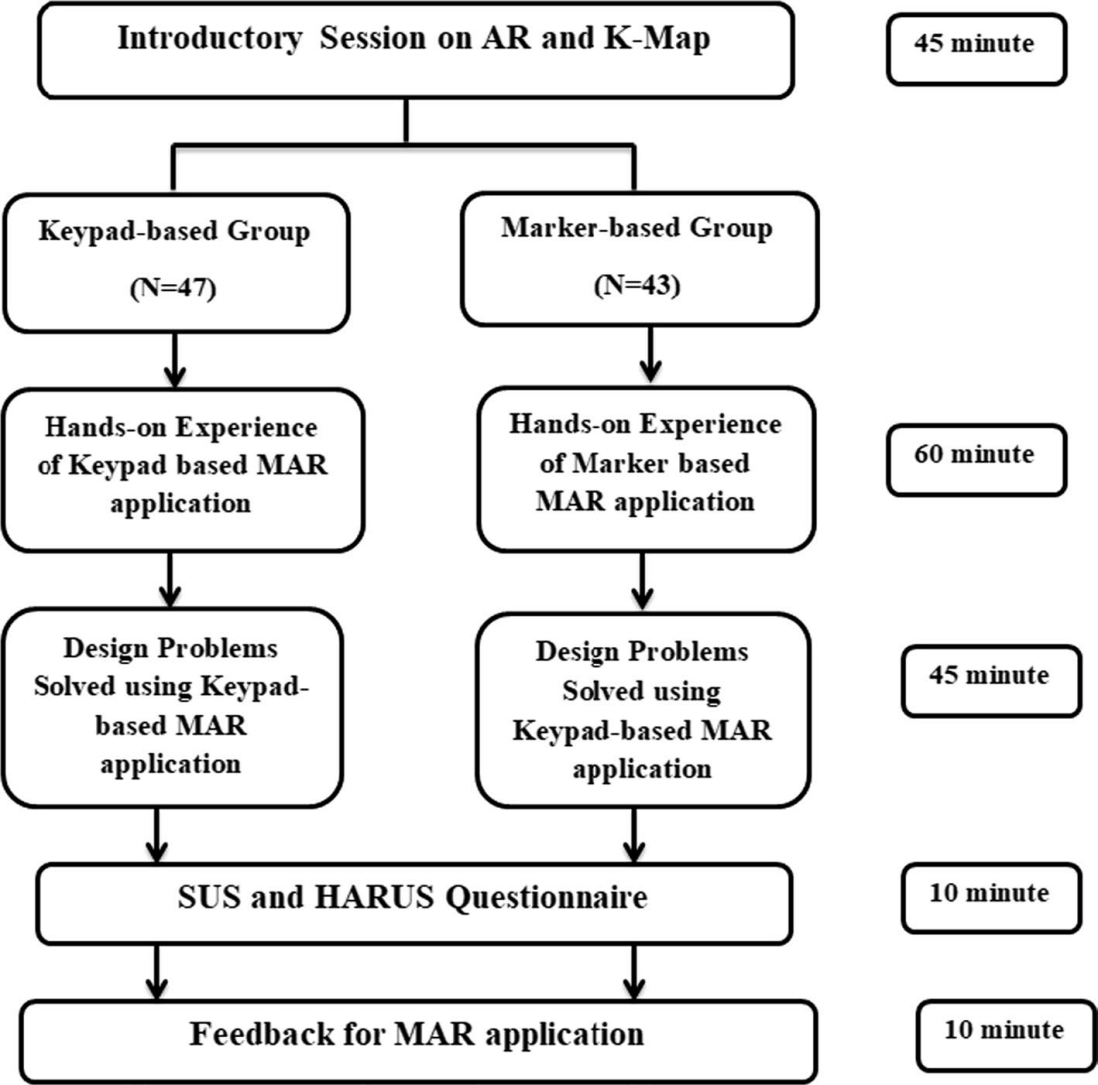
Experimental design

**Fig.17:**
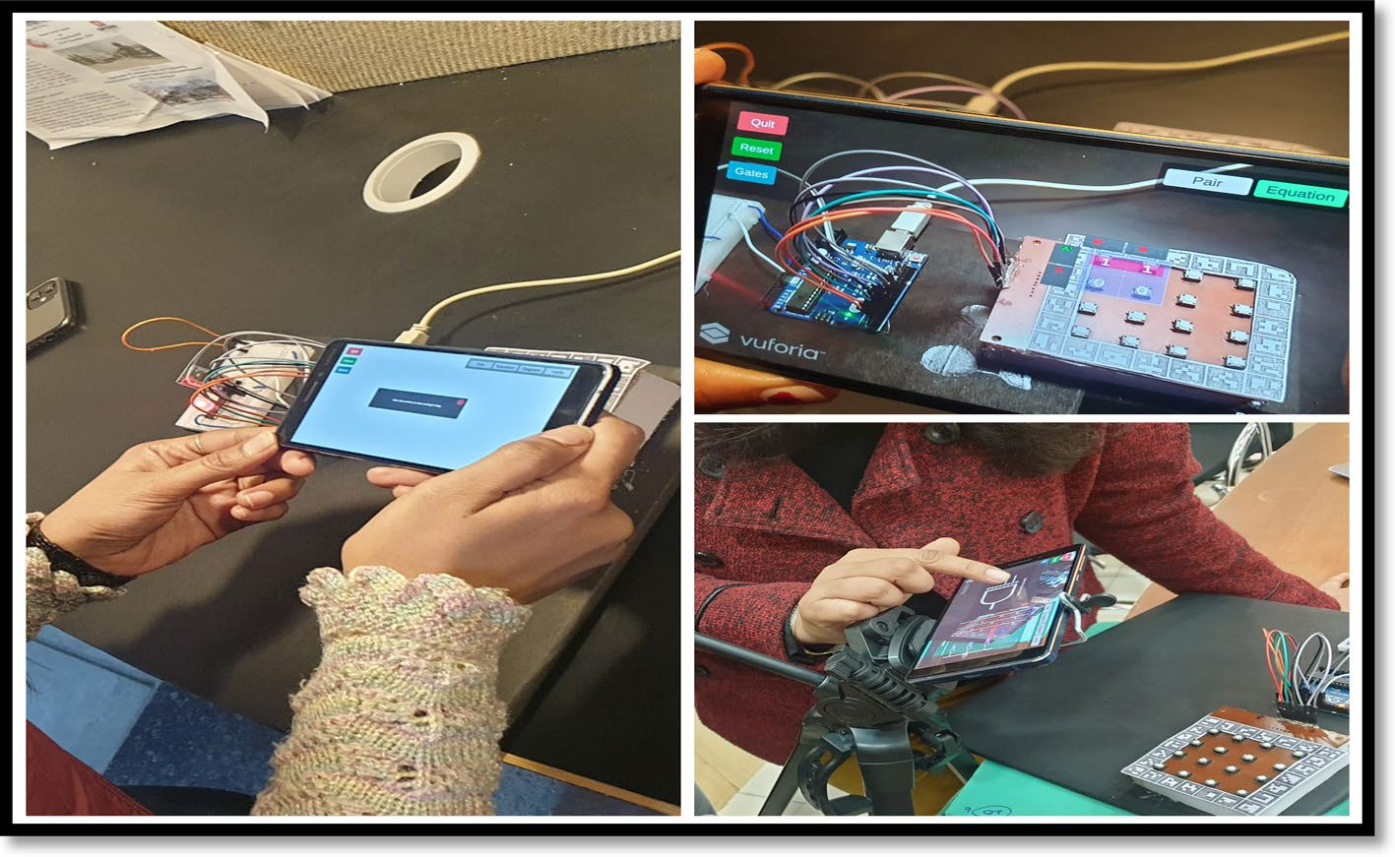
MAR using keypad matrix

**Fig.18:**
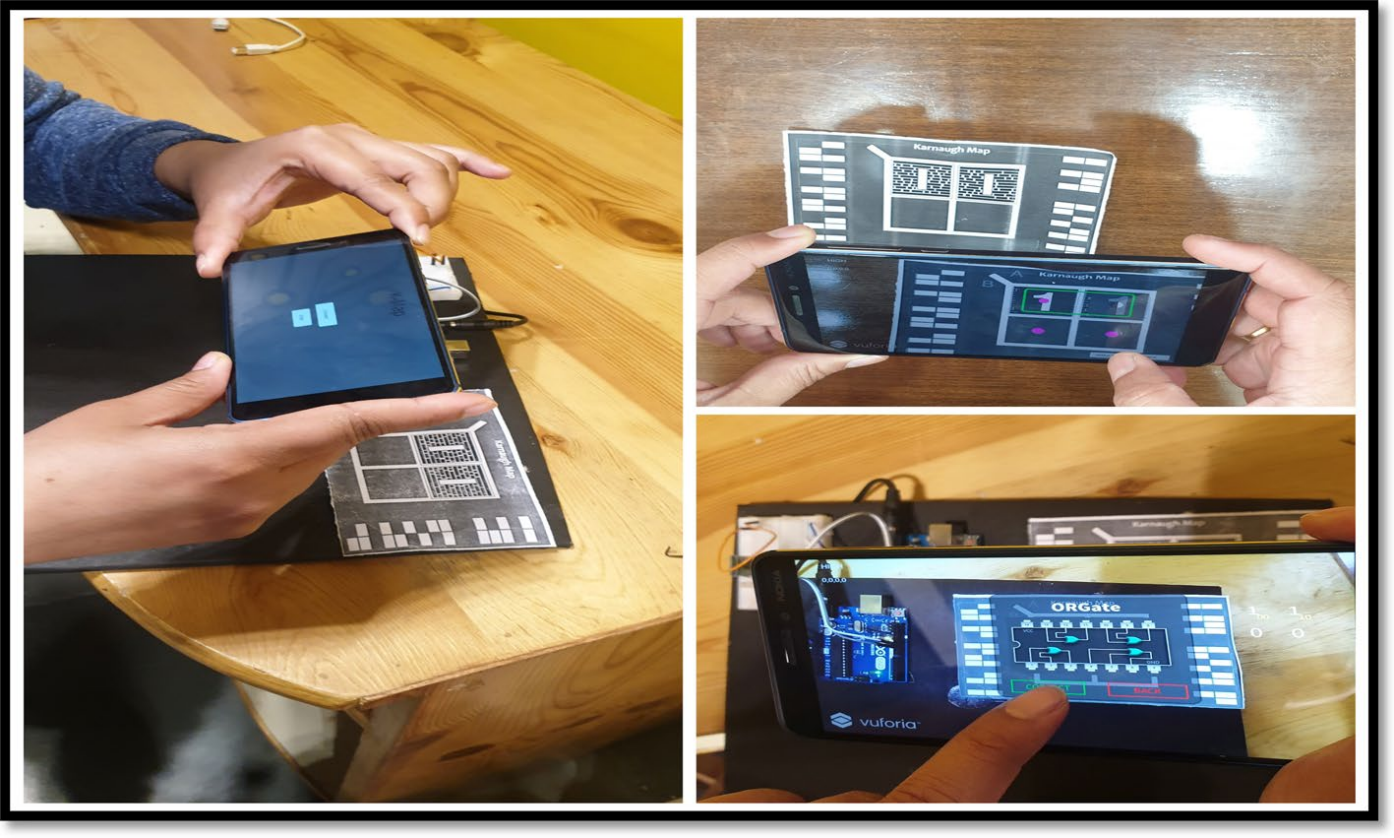
MAR with multiple markers

An experimental study was planned with the engineering students to evaluate the system usability of two different MAR applications developed. On Day 1, students using a keypad-based system were evaluated using SUS and HARUS models. On Day 2, students using multiple markers were evaluated using SUS and HARUS models. Figure 16 explains the experimental design of the study. The following activities were conducted during the evaluation of two different MAR applications:

- An introductory lecture about AR technology and K-maps was given to the students. The lecture lasted for 45 min.
- After that, depending upon the group, they were introduced to the different MAR applications. On Day 1, students were given the experience of using the MAR application with keypad matrix, as mentioned in Fig. 17. On Day 2, students were given the experience of using the MAR application with multiple markers, as illustrated in Fig. 18.
- After understanding the MAR application, five problem statements related to digital electronics circuits were assigned and asked them to use the MAR application to solve the circuit design problem and verify their solutions.
- Finally, the participants were asked to undertake the SUS and HARUS questionnaire about the MAR application and comment on the developed system's strengths and weaknesses.

## Result and discussion

The data were collected from SUS and HARUS questionnaires and were analyzed to determine the system usability of two MAR applications.

### SUS analysis

Even though the majority (63%) of respondents had never used AR before, they felt the framework to be easy and intuitive to use. SUS questionnaire constitutes positive meaning for the odd-numbered questions and negative for even-numbered. Table 7 presents the SUS questionnaire analysis, including mean values for both the groups: MAR using keypad matrix and MAR using multiple markers. The following steps were followed to calculate the SUS score.

- *Step 1* Evaluate the mean of the responses asked against each question.
- *Step 2* Subtract one from the collected user responses to calculate system usability for questions 1, 3, 5, 7, and 9. For example, if the average score is 4.49, subtract 1 from that to derive the new score of 3.49.
- *Step 3* Subtract the responses from 5 to calculate system usability for questions 2, 4, 6, 8, and 10. For instance, if the received score is 1.90, then subtract it from 5 to derive the new score of 3.10
- *Step 4* Sum the entire mean values and multiply them with 2.5 to obtain the final SUS score.

**Table 7:** Response of SUS questionnaire for keypad-based and marker-based AR system

S. no	Question	Keypad-based MAR	Multiple marker-based MAR
Q1	“I think that I would like to use this system frequently.”	3.43	2.00
Q2	“I found the system unnecessarily complex.”	3.34	2.94
Q3	“I thought the system was easy to use.”	3.40	2.16
Q4	“I think that I need the support of a technical person to be able to use the system.”	3.83	2.24
Q5	“I found the various functions in this system were well integrated.”	3.36	2.00
Q6	“I thought there was too much inconsistency in the system.”	3.30	2.94
Q7	“I would imagine that most people would learn to use this system very quickly.”	3.30	1.93
Q8	“I found the system very cumbersome to use.”	3.40	2.57
Q9	“I felt very confident using the system.”	3.43	1.86
Q10	“I needed to learn a lot of things before I could get going with this system.”	3.04	1.97
Total SUS score		33.83 * 2.5	22.61 * 2.5
		84.57	56.52

If the calculated SUS score is found to be greater than 55%, then the system's usability is acceptable (Brooke, 2020).

- *RQ1* To determine the system usability of marker-based and keypad-based AR application using the SUS model.

The overall SUS percentage for the keypad matrix group was 84.57% and for the multiple markers group was 56.52%. From the results, it could be concluded that the keypad approach was effective than multiple markers. The following may be the reasons;

- As per the SUS questionnaire, questions 1, 3, 5, 7, and 9 are related to ease of use, speed, and satisfaction. Table 7 presents both systems' responses, which indicate that the keypad-based system had a higher mean value for all the questions than multiple markers.
- Q 2, 4, 6, 8, and 10 are related to information inconsistency and system design. The received responses keypad-based system shows high consistency in the information than multiple marker systems

### HARUS analysis

Table 8 incorporates a HARUS questionnaire that evaluates usability in terms of manipulability and comprehensibility. In Table 8, Q1 to Q8 incorporate statements related to manipulability, and Q9 to Q16 relate to comprehensibility. Moreover, it contains users' responses for the two MAR variants (MAR using keypad matrix and MAR using multiple markers) on a 5-point Likert scale (1 corresponds to 'strongly disagree' and 5 corresponds to 'strongly agree'). The mean score of all the questions was analyzed and compared, as tabulated in Table 8.

- *RQ2* To compare and analyze system usability of marker-based and keypad-based AR application using the HARUS model.

**Table 8:** Responses to the HARUS questionnaire for keypad-based and marker-based AR systems

Parameters		Questions	Keypad-based MAR	Multiple marker-based MAR
Manipulability	Q1	“I think that interacting with this application requires a lot of mental effort.” (R)	4.26	3.42
	Q2	“I thought the amount of information displayed on the screen was appropriate.”	4.42	3.63
	Q3	“I thought that the information displayed on the screen was difficult to read.” (R)	4.05	3.57
	Q4	“I felt that the information display was responding fast enough.”	4.00	2.94
	Q5	“I thought that the information displayed on the screen was confusing.” (R)	4.63	4.15
	Q6	“I thought the words and symbols on screen were easy to read.”	4.10	3.52
	Q7	“I felt that the display was flickering too much.” (R)	4.26	3.63
	Q8	“I found the system very cumbersome to use.”	2.89	3.94
Comprehensibility	Q9	“I think that interacting with this application requires a lot of body muscle effort.” (R)	4.57	4.10
	Q10	“I felt that using the application was comfortable for my arms and hands.”	4.05	3.94
	Q11	“I found the device difficult to hold while operating the application.” (R)	4.00	3.37
	Q12	“I found it easy to input information through the application.”	4.31	3.31
	Q13	“I felt that my arm or hand became tired after using the application.” (R)	4.26	3.84
	Q14	“I think the application is easy to control.”	4.05	3.78
	Q15	“I felt that I was losing grip and dropping the device at some point.” (R)	4.63	4.47
	Q16	“I think the operation of this application is simple and uncomplicated.”	4.21	3.89

An independent sample *t* test was used to determine the significant difference between the system usability of keypad-based MAR and multiple marker-based MAR using the HARUS model. The normality of the data was checked before applying the *t* test on HARUS score. Table 9 presents the *t* test statistics; the mean score of the keypad-based group was 4.17 and for the marker-based group was 3.72 with a *p* value < 0.003, which indicates that the usability of the keypad-based MAR system is better as than the marker-based MAR system. During the interaction with students, they have mentioned that the keypad-based MAR application allows the user to select multiple K-map variables due to which it has better user interaction compared to the marker-based MAR application. They have also mentioned that the flickering effect is more in marker-based MAR applications due to which virtual content is not stable on the mobile screen. These are the key reasons due to which the keypad-based MAR application has a better HARUS score.

**Table 9:** *t* test analysis of HARUS scores

Group	N	Mean	S.D	***T***	***df***	***p ***value	95% confidence interval of the difference	
							Lower	Upper
Keypad-based group	47	4.17	0.403	3.26	88	0.003	0.169	0.730
Marker-based group	43	3.72	0.374					

### Participants opinion about AR

After the hands-on experience with MAR applications, the students were asked to share their experiences and feedback. Table 10 presents the students’ comments on the strengths and weaknesses of the MAR system. The students suggested that the game-based learning approach could be a milestone in engineering education as it is interactive and thus engages the users. They suggested improving the system graphics and design to improve the AR system.

**Table 10:** Students’ comments on strengths and weaknesses of the two MAR applications

System	Strengths	Weaknesses
Keypad-based MAR	Good innovation for future engineering education System acts as a self-guiding tool and helps us in correcting our answers Good approach for self-evaluation It helped to understand k-map, which is a complex topic that is hard to grab through traditional teaching It transforms the traditional teaching to game-based teaching	Enhance the UI of the application so that interaction could be easy with pushbuttons
Multiple Marker-based MAR	Provides end to end knowledge of K-Map Self-guiding to understand complex topics Could enhance the learning skills of the students by transforming learning into game-based learning	System is challenging to manage. If the paper is folded, then the system does not work properly Provides a lot of flickering effects

## Conclusion

Augmented reality technology encourages students to be self-learner, fosters a desire to explore new possibilities, and replaces costly laboratory equipment with multimedia models (Majeed & Ali, 2020; Noroozi et al., 2019; Singh et al., 2019; Wang et al., 2018). It creates an engaging environment by overlaying contextual information on real objects and further enhances their visual perception. Furthermore, integrating other technologies such as the Internet of things, machine learning, and artificial intelligence with AR-based learning systems aids in the creation of future learning systems. AR and VR-based learning tools allow more interaction between teachers and students and between the students and instructional content. As a result, it promotes an engaging environment because the instructor receives instant input from every student linked to the system, and every learner has regular access to the learning system.

In this study, two mobile AR-based applications were developed, and their system usability was measured using the SUS and HARUS models. An experimental study was conducted with the students to determine the usability of two AR applications, viz. MAR using multiple markers, and MAR using a keypad matrix. The SUS and HARUS analysis suggest that MAR application using keypad matrix has better-perceived usability (84.57%), manipulability, and comprehensibility (overall mean score = 4.17). After experiencing both systems, the participants found that the keypad-based AR system was more beneficial in the following aspects: information manipulation, ease of use, better interaction, and information accuracy. They also found the system more engaging while learning complex topics such as K-map. K-map is the base of digital electronics, and which further helps in circuit design in electronics engineering. So, as per the feedback, the keypad-based AR system could be utilized to teach concepts of K-Map to the engineering students.

During the AR session, it was observed that participants were excited to use AR technology, and they were keen to know about the developed AR systems. Overall, students' familiarity with AR technology and its usability, particularly in day-to-day learning, was the significant outcome of the study. The participants suggested scope of improvements such as enhancing the system's design, enlarging the graphics for better visualization, and a closed-loop feedback system from the hardware to validate the design. Students have also suggested developing AR-based systems to teach other subjects like transformers in electrical engineering, engineering graphics, circuit theory, and many more.

During the COVID-19 pandemic, AR and VR-based learning environments can also help the students and teachers to teach effectively. It was also observed that during the online teaching during the COVID-19 pandemic, students could not take laboratory courses to perform experiments on sophisticated machines and instruments. So, it is recommended that AR and VR technology can be utilized to develop immersive and interactive learning systems that provide real-world experience to the students during online teaching. Teaching complex and logical topics through such an interactive system may enhance learning outcomes, knowledge gain, critical thinking ability, and students' memory retention. As a beginner, the teacher could face technological and pedagogical issues related to the AR-based system but after spending some time understanding the technological background and stepwise working of the system. Educators can enhance the quality of their instruction and delivery. As a result, students will also be inspired and motivated to learn.

## Data Availability

Yes.

## Abbreviations

AR: Augmented reality
AOI: AND OR inverter
HARUS: Handheld Augmented Reality Usability Score
ICT: Information and Communication Technology
MAR: Mobile augmented reality
MR: Mixed reality
K-Map: Karnaugh map
POS: Products of sum
SUS: System usability scale
SOP: Sum of products
UEQ: User Experience Questionnaire
VR: Virtual Reality
